# Trunk appearance perception scale for physicians (TAPS-Phy) - a valid and reliable tool to rate trunk deformity in idiopathic scoliosis

**DOI:** 10.1186/s13013-016-0085-8

**Published:** 2016-08-17

**Authors:** Antonia Matamalas, Elisabetta D’Agata, Judith Sanchez-Raya, Juan Bago

**Affiliations:** 1 Vall d’Hebron Hospital, Passeig Vall d’Hebron, 119-129, 08035 Barcelona, Spain; 2 Vall d’Hebron Research Institut, Passeig Vall d’Hebron, 119-129, 08035 Barcelona, Spain

**Keywords:** Idiopathic scoliosis, Trunk deformity, Trunk appearance perception scale (TAPS), Reliability, Validity

## Abstract

**Background:**

Evaluation of trunk deformity by physicians in patients with idiopathic scoliosis (IS) has been considered an important part of clinical practice. Different methods to quantify the severity of trunk deformity by external observation have been reported. A valid tool to evaluate patients’ perception of trunk deformity, the Trunk Appearance Perception Scale (TAPS), is hereby validated for use by physicians (TAPS-Phy).

**Methods:**

Cross-sectional study of patients with non-surgically treated IS. Patients were prospectively recruited. On the day of the visit, a posterior-anterior radiograph in standard position and clinical photographs in three different views (anterior, posterior and forward bending position) were obtained. Patients also completed a TAPS questionnaire (TAPS-Pat). Three different observers scored the TAPS questionnaire (TAPS-Phy), based on the digital photographs previously obtained, twice a week. The angle of trunk inclination (ATRI) was also measured on digital photographs. Inter and intra-rater reliability was calculated through weighted kappa coefficient. External validity was tested by the Spearman correlation coefficient between the TAPS-Phy score and the scoliosis magnitude determined using the magnitude of the largest curve (MLC), ATRI, and TAPS-Pat.

**Results:**

Fifty two patients (46 women; mean age 16.6 years) were included. The average curve magnitude of the major curve was 44°. Mean scores of TAPS-Phy for the three evaluators ranged from 3.4 to 3.5. No differences between the three means were found. TAPS-Phy showed good internal consistency (Cronbach’s alpha coefficient 0.84). Inter-observer reliability ranged from slight to substantial (0.14 to 0.63); intra-observer reliability ranged from 0.35 to 0.99. Correlation between TAPS-Phy and ATRI (*r* = −0.54 to −0.75), MLC (*r* = −0.47 to −0.6) and TAPS-Pat (*r* = 0.29 to 0.34) were statistically significant (*p* < 0.01).

**Conclusions:**

TAPS-Phy is a valid and reliable scale to rate a physician’s impression of the severity of the deformity in patients with idiopathic scoliosis and can be useful in routine clinical records.

**Electronic supplementary material:**

The online version of this article (doi:10.1186/s13013-016-0085-8) contains supplementary material, which is available to authorized users.

## Background

Trunk deformity is a crucial component of idiopathic scoliosis (IS). Physicians’ impressions of the severity of trunk deformity could be of interest for the clinical record. Different methods have been used to quantify trunk deformity by external evaluators. Theologis et al. [1] assessed the validity of a Cosmetic Spinal Score: an external evaluator quantified the severity of the trunk deformity from 1 to 10. This score showed a moderate inter and intra-observer reliability; the correlation between the score and the Cobb angle was moderate (*r* = 0.46) whereas the correlation with the rib hump (assessed by ISIS Scan) was *r* = 0.63. Raso et al. [2] asked several external evaluators to rate 8 components of the trunk deformity on a scale from 0 to 50. The correlation between an evaluator’s score and Cobb angle was 0.41. Data about inter- and intra-observers’ reliability were not provided. In addition, the external validity of some of these characteristics of the deformity has not been demonstrated. Finally, Zaina et al. [3] developed the Trunk Aesthetic Clinical Evaluation (TRACE) tool to evaluate four aspects of the severity of the trunk deformity: shoulder, scapula, hemi-thorax, and waist asymmetry. To assist the external evaluator to rate each of these features, the tool provides clinical photographs of patients with progressive degrees of deformity. The inter-observer reliability, assessed by unweighted kappa coefficient, was poor and highly variable (kappa ranged from 0.09 to 0.14).

The Trunk Appearance Perception Scale (TAPS) is a validated instrument to test the trunk deformity perceived by the patient [4]. The scale includes three sets of drawings that correspond to the three views of the trunk: from the back, from the front and in forward bending position (Adams test). The patient has to choose the picture that seems more appropriate to the perception of his/her own image. A moderate correlation (*r* = −0.55) between TAPS score and the radiological magnitude has been reported. We hypothesized that the TAPS scale completed by the physician while performing a physical examination could be a method to quantify and describe the severity of the trunk deformity. This research is aimed to validate TAPS completed by physicians (TAPS-Phy) and to compare these data with TAPS fulfilled by patients (TAPS-Pat).

## Methods

This is a prospective cross-sectional study evaluated and approved by the Ethics and Clinical Research Committee from Hospital Vall d’Hebron. Patients with idiopathic scoliosis consulting in the out-patients clinic of our institution and who met the inclusion criteria where consecutively recruited. Inclusion criteria for this study were: diagnosis of idiopathic scoliosis, aged between 10 and 40 years old, receiving non-operative treatment (either brace or observation), and patient consent to participate. The sample was stratified according to the radiological magnitude, measured with Cobb angle, of the major curve in four groups of 13 patients: <30°, 30° to 45°, 45° to 60°, and >60°.

### Radiographic measures

For each patient, a postero-anterior radiograph of the full trunk in the standing position was taken. An experienced clinician (author AM) took all measurements using digital software (Surgimap Spine Software® Nemaris Inc, New York, United States). The coronal Cobb angle of the proximal thoracic (PT), the main thoracic (MT) and the thoracolumbar/lumbar (TL/L) curves were measured. The magnitude of the largest curve (MLC) was also used for statistical analysis.

### Photographic measures

Clinical photographs were taken of each patient with a F2.8 LUMIX digital camera in a standardized manner on the same day of the visit by a single trained examiner (author ED). For each of the cases, photographs from the back and front in standing position were obtained. To evaluate the transverse plane deformity, a photograph, taken from the head of the patient, adopting a forward bending position (Adams test), was also obtained.

**Fig. 1:**
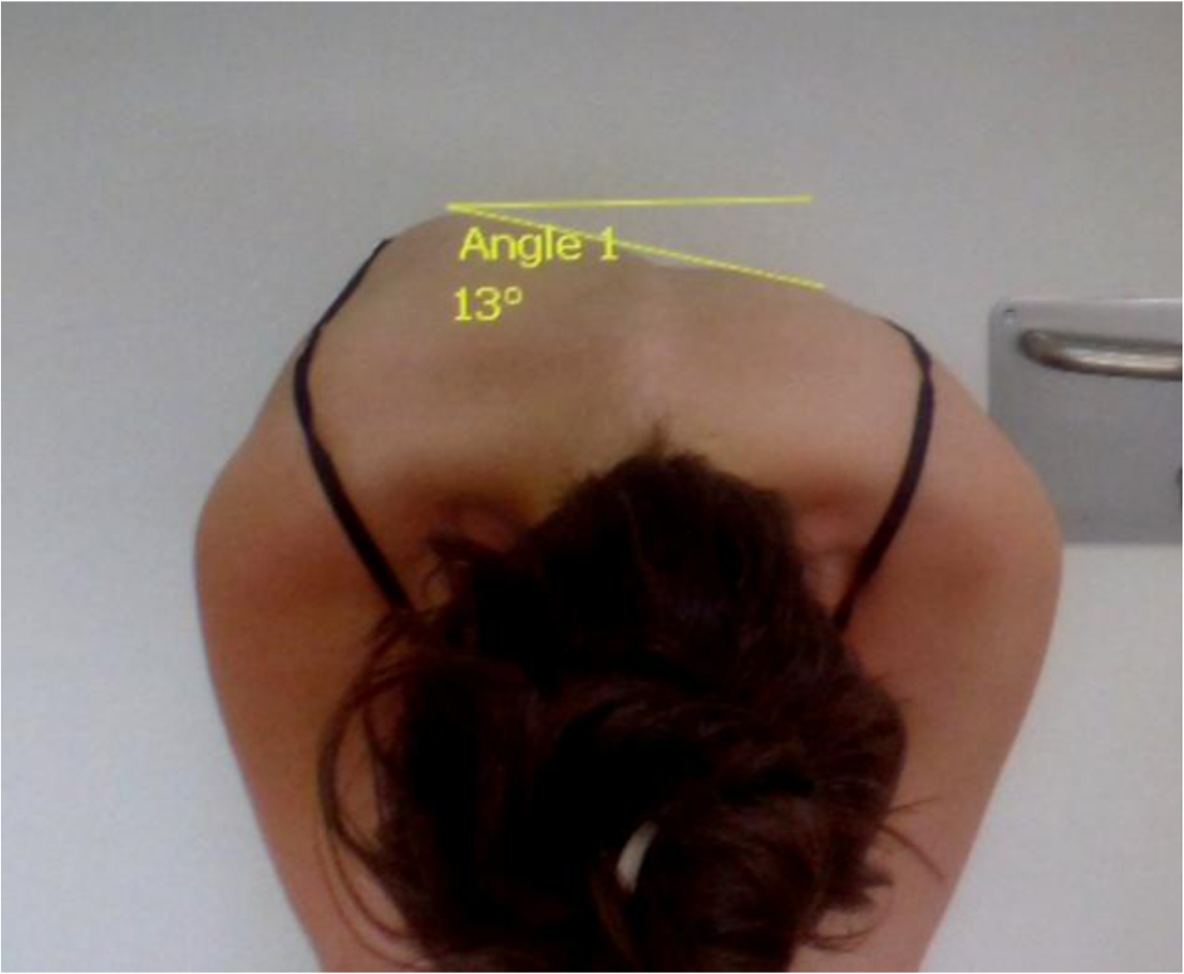
Angle of Trunk Inclination measured on digital photography in forward bending position. Method used to measure the angle

On the photographs, the angle of trunk inclination (ATRI) was measured using Surgimap software. The photographic ATRI angle was defined by the angle between a line connecting the uppermost points of the left and right posterior rib cage, with the horizontal line (Fig. 1).

### Questionnaire

All of the patients completed the TAPS questionnaire (TAPS-Pat). The TAPS scale includes three sets of drawings that correspond to the three views of the trunk: from the front, from the back and in a forward bending position (Additional file 1). Each drawing is rated from one (worst deformity) to five (no deformity) and an average score (sum of the values of the three drawings divided by three) of between one and five is obtained.

The same questionnaire was recorded by observers. Three observers, with different degrees of experience in scoliosis (a rehabilitation physician, an orthopedic surgeon and a psychologist), completed the TAPS-Phy tool, scoring the clinical photographs of the patients. This procedure was performed on two occasions, one week apart.

### Statistical analysis

We used descriptive statistics including mean and standard deviation. The non-parametric Kruskal-Wallis-Test was used to compare each mean TAPS score of the three observers. Internal consistency of TAPS-Phy overall score was tested by pooling data of the first measurement from the three observers and calculating Cronbach’s alpha coefficient.

To test inter and intra-rater reliability, the weighted kappa coefficient was calculated. According to Landis and Koch [5], the kappa coefficient agreement was considered as: slight (0.01 to 0.20), fair (0.21–0.40), moderate (0.41–0.60), substantial (0.61 to 0.80) and almost perfect (0.81 to 0.99). External validity of TAPS-Phy was tested by the Spearman correlation coefficient between TAPS-Phy score and scoliosis MLC, ATRI and TAPS-Pat. SPSS 18.0 statistical software was used for data analysis. Statistical significance was set at *p* = 0.05.

## Results

### Descriptive analysis

Fifty two patients with IS were included (six men and 46 women); the mean age was 16.6 years (range ten to 37 years) and the average MLC was 44° (range 20° to 76°). The average PT was 20° ± 15; the average MT was 41° ± 16.8 and the average TL/L was 33° ± 15.5. Scoliosis pattern involved a single curve in 31 cases and double curve in 21 cases.

Mean scores of TAPS-Phy for the three evaluators were 3.4 (±0.7), 3.4 (±0.8) and 3.5 (±0.5). These differences were not statistically significant (*p* = 0.6). Average pooled TAPS-Phy scores for the three evaluators was not different between single and double curves (*p* > 0.1). The average of TAPS-Pat was 3.2 (±0.9). In Table 1, the coefficients of agreement for each pair of observers are specified. The coefficients ranged from 0.14 to 0.63. Cronbach’s alpha coefficient, used to assess TAPS-Phy’s internal consistency, was 0.84.

**Table 1:** Inter-observer weighted kappa coefficient for each pair of observers, each TAPS-Phy item, and total score. All values achieved statistical significance (*p* < 0.05)

		Observer 2	Observer 3
TAPS-Phy 1	Observer 1	0.42	0.31
	Observer 2		0.14
TAPS-Phy 2	Observer 1	0.63	0.30
	Observer 2		0.50
TAPS-Phy 3	Observer 1	0.36	0.32
	Observer 2		0.36
TAPS-Phy total	Observer 1	0.50	0.40
	Observer 2		0.40

In Table 2, the intra-observer kappa coefficients are specified for each observer. Observer 1 had substantially higher values (kappa coefficient ranging from 0.87 to 0.99) than the other two observers.

**Table 2:** Intra-observer weighted kappa coefficients for each observer and each TAPS-Phy item and TAPS-Phy total score. All values achieved statistical significance (*p* < 0.01)

	Observer 1	Observer 2	Observer 3
TAPS-Phy 1	0.93	0.48	0.37
TAPS-Phy 2	0.91	0.52	0.63
TAPS-Phy 3	0.87	0.56	0.35
TAPS-Phy total	0.99	0.43	0.41

### Construct validity

In Table 3, the correlation coefficients of TAPS-Phy and item two of TAPS-Phy (transverse plain view) with ATRI were detailed for each observer. All correlations achieved statistical significance. The correlation coefficients between TAPS-Phy and MLC were statistically significant for all the three observers (Observer 1 *r* = − 0.6, Observer 2 *r* = − 0.52, Observer 3 *r* = − 0.47; p-value < 0.001). Finally, the correlations between TAPS-Phy scores and TAPS-Pat were respectively: Observer 1 = 0.34 (*p* < 0.01), Observer 2 = 0.33 (*p* = 0.025), Observer 3 = 0.29 (*p* < 0.05).

**Table 3:** Spearman correlations coefficients between ATRI and TAPS-Phy total score and TAPS-Phy item 2. All values achieved statistical significant (*p* < 0.001)

	Observer 1 ATRI	Observer 2ATRI	Observer 3ATRI
TAPS-Phy 2	−0.54	−0.75	−0.68
TAPS-Phy Total	−0.50	−0.58	−0.57

## Discussion

When evaluating a patient with IS, the subjective physician’s impression of the patient’s trunk appearance may be interesting data to collect for the clinical record. We felt that this goal might be accomplished if physicians completed the Trunk Appearance Perception Scale (TAPS). TAPS is a validated tool typically used to assess the patient’s perception of trunk deformity. Our objective is to test the validity of TAPS as scored by physicians (TAPS-Phy).

TAPS-Phy has good internal consistency (Cronbach’s alpha = 0.84), similar to that published for TAPS as scored by patients (Cronbach’s alpha = 0.89) (5). The inter-observer reliability ranged from 0.16 (Item 1 Obs 2 vs Obs 3) to 0.63 (Item 2 Obs 1 vs Obs 2). For the total score (TAPS-Phy total), reliability ranged from 0.4 to 0.5 between the different pairs of observers and the degree of agreement can be considered to be moderate. The intra-raters’ reliability ranged from 0.37 (Obs 3) to 0.93 (Obs 1). For the total score, kappa weighted coefficients were 0.99, 0.43 and 0.41 respectively. Interestingly, Observer 1 reached an almost perfect agreement. As she is the most expert in the management and physical examination of patients with idiopathic scoliosis, this finding could indicate that the reliability of TAPS-Phy would be influenced by the observer’s expertise, which probably warrants investigation.

The use of the kappa coefficient as a statistical test to assess the reliability deserves some consideration. TAPS should be considered an ordinal scale, so the kappa coefficient test is adequate to evaluate inter and intra observer reliability. As we were more interested in the agreement across major categories in which there is meaningful difference, we determined the weighted kappa, which assigns less weight to agreement as categories are further apart. Moreover, the use of the kappa coefficient as a measure of the observer variation in clinical practice has been questioned [6]. We could note the contradictory results for the inter-observer reliability as a sign of the difficulty of interpreting the kappa coefficient. The TAPS-Phy inter-rater kappa coefficient for the overall score ranged between 0.4 and 0.5 (moderate) whereas Cronbach’s alpha coefficient, which coincides with intraclass correlation coefficient, was 0.84 (substantial).

TAPS-Phy has been proved to be a valid test since the correlation with the Cobb angle varies between −0.47 and −0.6. Interestingly, the observed correlation between the TAPS-Pat and MLC was −0.41, slightly lower than the average observed for external evaluators. The relationship between the questionnaire and ATRI measured in clinical photography was also evaluated. The correlation with TAPS-Phy total score was moderate (rho ranged between −0.50 and −0.58), while the correlation with the TAPS-Phy item2, evaluating the transverse plane deformity, was moderate to substantial (rho ranged between −0.54 and −0.75). Therefore, the external evaluator’s perception of the deformity in the transverse plane has a good correlation with the clinical deformity measured by the photography. However, the reliability of ATRI measurement with digital photography has not been previously studied and therefore representing a limitation to our study. A priori, problems with standardization of patient and camera positioning can be anticipated owing to low reliability. On the other hand, we did not have the ATRI measured with an inclinometer, which is the recommended method of measurement [7]. Finally, the correlation between TAPS-Phy and TAPS-Pat ranged from 0.29 to 0.34. Although statistically significant, this correlation was unexpectedly low, and suggests that the perceptions of trunk appearance between patients and physicians may vastly differ. Similarly, Rigo et al. also found a discrepancy between the TAPS completed by patients and their parents [8]. Also, the correlation between the clinical and radiological deformity and the external evaluator’s perception is higher than that observed for the patients. All these data probably suggest that the subjective perception of patients is influenced by several factors that are not perceived by external evaluators. The idea of an external evaluator using a reference instrument for rating trunk deformity comes from the work of Zaina et al. [3]. They designed the Trunk Aesthetic Clinical Evaluation (TRACE) tool to rate four aspects of the trunk deformity: shoulder, scapula, hemi-thorax, and waist asymmetry. For each of these areas, the tool includes clinical photographs of patients with progressive degrees of deformity. Unfortunately, they report poor and highly variable inter-observer reliability (kappa coefficient from 0.09 to 0.14). However, they used the unweighted kappa coefficient that evaluates the agreement regardless of the order of categories. Moreover, it is unclear if the photos published in the initial work should be the only ones to be used or if each separate center could use their own photos. Conversely, TAPS is easily accessible, and according to its metric properties, appears to be a reliable and valid instrument.

However, the use of TAPS-Phy presents several limitations. Given the low intra- and inter-observer agreements shown in our study, we reject our hypothesis that it represents a useful system. Furthermore, the correlation between TAPS-Phy and the magnitude of scoliosis was only fair to moderate. These data indicate that the discriminant validity of the instrument is limited and question the capability of TAPS-Phy to discriminate between patients with different curve magnitude.

## Conclusions

While TAPS- Phy was shown to be a valid and reliable scale with promising clinical utility in rating a physician’s impression of the severity of the deformity in patients with idiopathic scoliosis, the metric properties identified in our study question its wider use as an estimator of the radiological magnitude of scoliosis.

## Additional file

Additional file 1: TAPS scale. Presentation of the scale. (PDF 210 kb)
