# Supplementary figures and images for: Trunk appearance perception scale for physicians (TAPS-Phy) - a valid and reliable tool to rate trunk deformity in idiopathic scoliosis
Source: Scoliosis Spinal Disord. 2016 Aug 17;11:24. doi: 10.1186/s13013-016-0085-8 (PMC4988037; doi:10.1186/s13013-016-0085-8)

**Annex 1: Trunk Appearance Perception Scale (TAPS)**

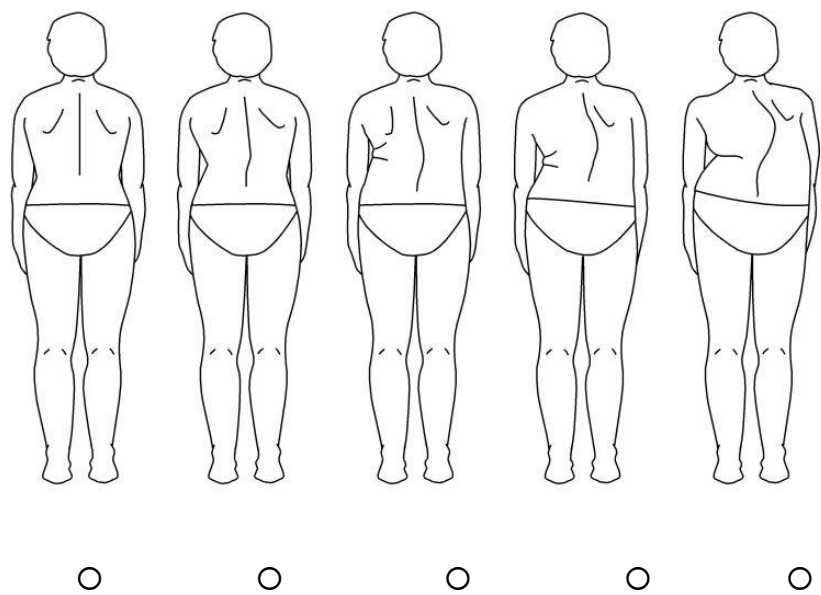

**Item 1**

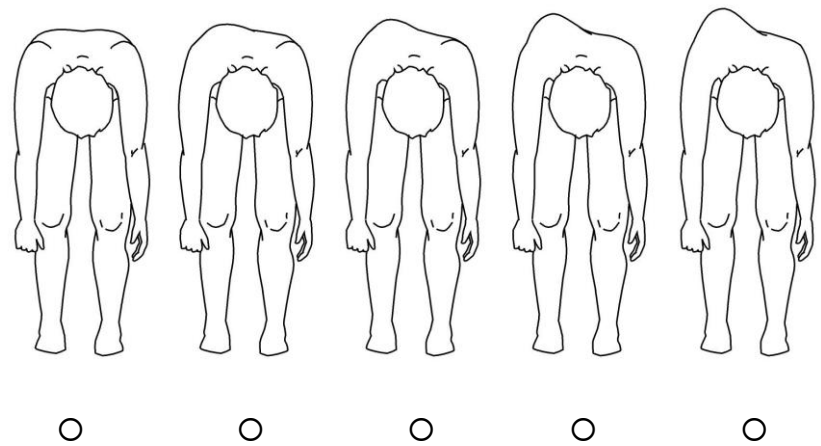

**Item 2**

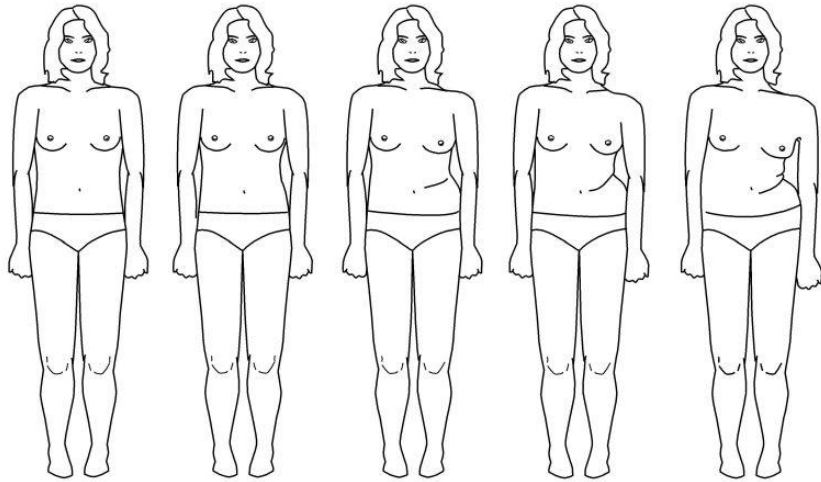

☐ ☐ ☐ ☐ ☐

**Item 3 (females)**

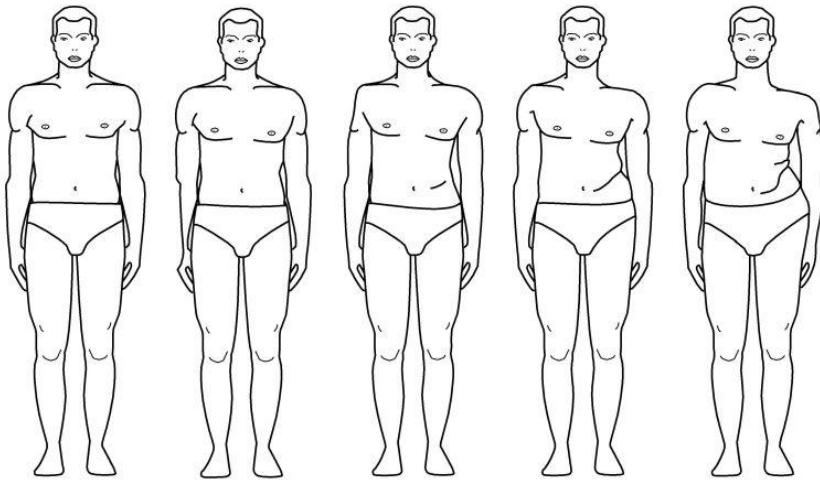

☐ ☐ ☐ ☐ ☐

**Item 3 (males)**

Supplement: Additional file 1: — TAPS scale. Presentation of the scale. (PDF 210 kb) [file 13013_2016_85_MOESM1_ESM.pdf]
